# The cameroon mobile phone sms (CAMPS) trial: a protocol for a randomized controlled trial of mobile phone text messaging versus usual care for improving adherence to highly active anti-retroviral therapy

**DOI:** 10.1186/1745-6215-12-5

**Published:** 2011-01-07

**Authors:** Lawrence Mbuagbaw, Lahana Thabane, Pierre Ongolo-Zogo, Richard T Lester, Edward Mills, Jimmy Volmink, David Yondo, Marie José Essi, Renée-Cecile Bonono-Momnougui, Robert Mba, Jean Serge Ndongo, Francois C Nkoa, Henri Atangana Ondoa

**Affiliations:** 1 Centre for the Development of Best Practices in Health(CDBPH), Yaoundé Central Hospital, Avenue Henri Dunant, Messa, PO Box 87, Yaoundé, Cameroon; 2 Department of Clinical Epidemiology and Biostatistics, McMaster University, Hamilton, ON, Canada; 3 Biostatistics Unit, Father Sean O'Sullivan Research Centre, St Joseph's Healthcare, Hamilton, ON, Canada; 4 Department of Medicine, Division of Infectious Diseases University of British Columbia, Vancouver, BC, Canada; 5 British Columbia Centre for Disease Control, Vancouver, BC, Canada; 6 Faculty of Health Sciences, University of Ottawa, Ottawa, ON, Canada; 7 Faculty of Health Sciences Stellenbosch University, Capetown, South Africa

## Abstract

**Background:**

This trial aims at testing the efficacy of weekly reminder and motivational text messages, compared to usual care in improving adherence to Highly Active Antiretroviral Treatment in patients attending a clinic in Yaoundé, Cameroon.

**Methods and Design:**

This is a single-centered randomized controlled single-blinded trial. A central computer generated randomization list will be generated using random block sizes. Allocation will be determined by sequentially numbered sealed opaque envelopes. 198 participants will either receive the mobile phone text message or usual care. Our hypothesis is that weekly motivational text messages can improve adherence to Highly Active Antiretroviral Treatment and other clinical outcomes in the control group by acting as a reminder, a cue to action and opening communication channels. Data will be collected at baseline, three months and six months. A blinded program secretary will send out text messages and record delivery.

Our primary outcomes are adherence measured by the visual analogue scale, self report, and pharmacy refill data. Our secondary outcomes are clinical: weight, body mass index, opportunistic infections, all cause mortality and retention; biological: Cluster Designation 4 count and viral load; and quality of life. Analysis will be by intention-to-treat. Covariates and subgroups will be taken into account.

**Discussion:**

This trial investigates the potential of SMS motivational reminders to improve adherence to Highly Active Antiretroviral Treatment in Cameroon. The intervention targets non-adherence due to forgetfulness and other forms of non-adherence.

**Trial Registration:**

Pan-African Clinical Trials Registry PACTR201011000261458

**http://clinicaltrials.gov/** **NCT01247181**

## Background

Mobile text messages using the short message service (SMS) are a cheap and non-invasive means of communication that can be used to convey health related messages to owners of mobile phones. There is contradictory evidence concerning the role of mobile phones in ameliorating health outcomes, especially in less developed countries where private ownership and use of mobile phones is not as widespread as in other more developed countries[1]. Currently, Africa has the greatest uptake of mobile phone technology [2].

The advent of Highly Active Antiretroviral Therapy (HAART) has markedly reduced morbidity and mortality associated with the Human Immune deficiency Virus (HIV) [3]. Much effort has been put into the scaling up of access to HAART [4]. The efficacy of HAART depends largely on compliance to treatment regimens. Poor adherence is associated with poor virological and immunological response. It is also responsible for the development of resistant strains [5]. Very high levels (> 95%) of adherence are necessary for sustained clinical success [6].

Our search for papers on the use of SMS technology to improve adherence to HAART revealed two protocols for trials in Kenya (WelTel Kenya 1) [7] and India (HIVIND) [8]. The WelTel Kenya 1 trial reported significant improvements in adherence and viral load [9].

The World Health Organization (WHO) has prioritized the use of new technologies to assist health delivery in resource-limited settings [10]. The SMS is already used for business transactions, personal communication, advertising and betting. There is a potential for new benefits to be discovered in the use of mobile phone technology in health interventions for resource- limited countries [11]. In South Africa, the SMS has been demonstrated to improve HIV health care service delivery by ameliorating communication between health workers and patients, and also as an appointment reminder [12].

Additionally, a Cochrane systematic review found that mobile phone calls and reminders can improve adherence to tuberculosis care [13]. Another study investigating the use of mobile phone technology to improve adherence to HAART discouraged the use of phone calls as they are time and labor intensive [14]. A third study, investigating the use of text messaging to improve adherence to primary care found that it was more cost effective than phone calls[15]. The cost of phone calls may also be a hindrance in developing countries. The Indian trial [8] is testing automated phone calls coupled to picture messages. This may reduce the time and labor costs involved. Another study reported high satisfaction with two way text messaging [16]. These findings suggest that the more feasible application of the mobile phone in health would be the SMS.

The goal of this trial is to assess whether sending weekly motivational text messages via a mobile phone versus no text messaging will improve adherence to HAART and clinical outcomes among HIV positive patients over a 6 month period. We hypothesize that sending one weekly motivational reminder will produce a change in behavior to enhance drug or treatment adherence and hence clinical outcomes. SMS-delivered interventions are capable of producing a positive behavior change [17].

Participants will be selected from the Accredited Treatment Centre (ACT) of the Yaoundé Central Hospital (YCH).

## Methods and Design

The trial is registered with the Pan-African Clinical Trials Registry http://pactr.org as PACTR201011000261458 and with clinical trials.gov as NCT01247181.

### Funding

Partial funding for this study was obtained from an international postdoctoral research fellowship awarded by the Canadian Institutes of Health Research (CIHR) HIV Clinical Trials Network (CTN) to the principal investigator.

### Study Design

**Figure 1:**
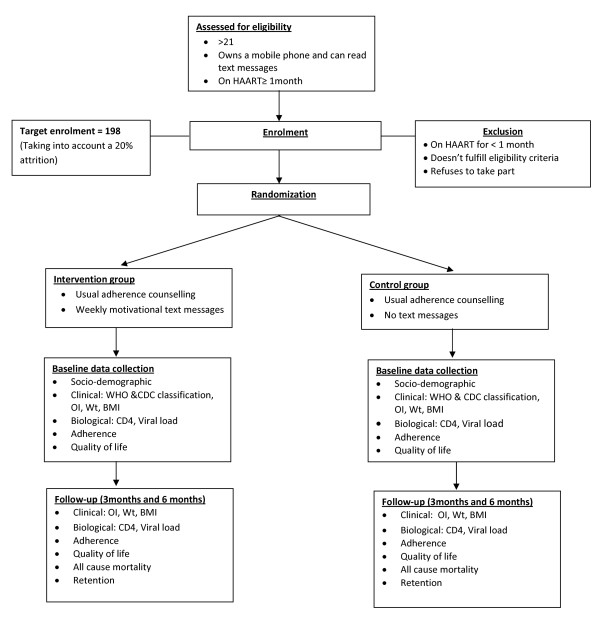
**Consolidated Standards of Reporting Trials-CONSORT diagram of study design**.

Using a 1:1 allocation ratio, patients at the Yaoundé Central Hospital (YCH) Accredited Treatment Centre (ATC) will be randomized to either receive a text message reminder to take their medication or not (Figure 1: Consolidated Standards of Reporting Trials-CONSORT diagram of study design). Both groups of patients will benefit from the usual care provided in this centre which includes adherence counseling and rarely, home visits.

### Randomization

This is a parallel group design evaluating the effects of adding weekly SMS text messages using mobile phones to usual care (intervention) versus usual care alone (control) among HIV positive patients on HAART. Eligible and consenting patients will be randomized to intervention and control arms using 1:1 allocation ratio by opaque sealed envelope method. A computer generated randomization list will be generated using random block sizes of 2, 4 and 6, by the Father Sean O'Sullivan Research Centre Biostatistics Unit at St Joseph's Healthcare/McMaster University. The allocation codes will then be put in sequentially numbered opaque sealed envelopes and administered by the trained Research Staff at YCH ATC centre. Trained interviewers - blinded to group allocation - will collect data using a pretested data collection form containing socio-demographic data, clinical information and adherence rates at baseline, 3 and 6 months. The data analyst will also be blinded to group allocation.

### Trial Setting

Cameroon is a sub-Saharan central African country, made of ten provinces and a population of 18 million inhabitants [18]. The Centre and capital province has a population of about 3.2 million inhabitants. The adult prevalence rate of HIV in the country is 5.1% [19]. Subjects will be recruited from the YCH ACT. This is an urban centre in the heart of the capital city, Yaoundé. The adult prevalence of HIV in the Centre province is 4.7%. The YCH is a tertiary level general teaching hospital with a capacity of 381 beds. It employs nearly 800 staff including 95 doctors and 270 nurses [20]. The ACT has a very high recruitment rate of approximately 40 new cases per week and caters for 6500 regular clients. It is the largest HIV clinic in the country and offers enormous potential for recruitment.

### Participants: inclusion/exclusion criteria

We will include subjects who are aged above 21 years, own a mobile phone and can read text messages, and who have been on HAART for at least a month. Informed consent is a prerequisite for participating in the study, and will be provided orally and in writing.

We will exclude participants who have been on HAART for less than a month, are aged less than 21 years. Participants who have used HAART for at least one month are chosen so that we can calculate a baseline figure for adherence.

### Intervention

**Figure 2:**
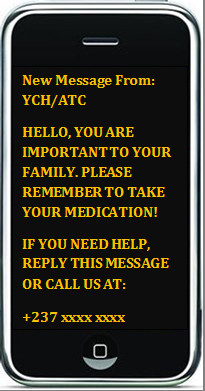
**Example of a text message**.

We will send a short text message to the participants in the intervention group in both French and English. The content of the message will be motivating and will act both as a reminder and a cue to action (Figure 2: Example of a text message). The message will also contain a phone number they can call back if they need help. The content will be varied so as to retain participants' attention throughout the period of the study and to explore the various aspects of behavior change. The program secretary will have a list of phone numbers to which he/she will send the messages every week and will use the 'delivery report' function to ensure that the messages have been delivered. One message will be sent per week in the morning of a chosen day. The average cost for text messages on any networks is 50 CFA Frs. CFA (≈0.1 USD). Text messaging will be provided as an add-on to usual care which includes regular HAART counseling and occasional home visits.

### Control

In the control arm, patients will receive the usual care provided to all patients of the ATC which includes regular HAART counseling and occasional home visits. They will be given a number to call if they have questions. They will not receive any text messages, but they will be interviewed at baseline, 3 months and 6 months.

### Study Objectives

#### Primary Objective

The primary objective of this trial is to investigate the effect of adding the SMS to usual care versus usual care alone in improving and maintaining adherence to HAART in HIV positive patients on HAART at 3 and 6 months. There are several methods used to evaluate or measure adherence to medications, each with advantages and disadvantages [21,22]. Thus, there is no gold standard in measuring medication adherence [21,23]. The common approach is to use multiple methods to compare or assess the robustness of the estimates of adherence. For this study, we will use three commonly used measures of adherence - namely Visual Analogue Scale (VAS), Pharmacy Refill Data (PRD), and Self Report (SR). We will use VAS as the primary method of measuring adherence. The VAS is highly correlated with more objective methods like using Microelectronic Monitoring System (MEMS) caps [24]. VAS method has also been widely used in several RCTs evaluating different interventions including mobile text messaging to enhance adherence to HAART [8,21]. PRD and SR will be used to fill any missing data on VAS.

#### Secondary objectives

The secondary objectives include comparing clinical outcomes such as weight, body mass index (BMI), opportunistic infections (OI), Cluster Designation (CD) 4 count, viral load and quality of life between the groups. These comparisons will be performed at 3 and 6 months.

### Outcome measures

Our primary outcome will be adherence rates, measured using VAS. SR and PRD will also be used to supplement VAS (Table 1).

**Table 1:** Overview of outcome measures

Outcome measures	Scale	Type	Measure	Analysis method
**Primary**				

Adherence at 6 months*				

VAS	Ordinal	Binary	VAS percentage >95%	Chi-squared test

Self report	Ordinal	Binary	% adherence in last month > 95%	Chi-squared test

PRD	Ordinal	Binary	% of complete refills >95%	Chi-squared test

**Secondary**				

Weight	Ratio	Continuous	Change in weight	T-test

BMI	Ratio	Continuous	Change in BMI	T-test

OI	Nominal	Binary	Occurrence of new OI	Chi-squared test

Mortality	Nominal	Binary	All deaths	Chi-squared test

Retention	Nominal	Binary	Number retained in care	Chi-squared test

CD4 count	Ratio	Continuous	Change in CD4 count	T-test

Viral load	Ratio	Continuous	Change in viral load	T-test

Satisfaction with care	Ordinal	Categorical	Change in satisfaction scores	T-test

QoL	Ordinal	Categorical	Change in QoL scores	T-test

VAS: visual analogue scale, PRD: pharmacy refill data, BMI, body mass index, OI: opportunistic infection, CD4: Cluster Designation 4, QoL: Quality of Life.

* All three measures of adherence will also be analyzed as continuous outcomes using T-test

Our Secondary endpoints will be;

• Clinical: Weight, BMI, opportunistic infections

• Biological: CD4 count, viral load

• Quality of life (QOL): Measured with the SF-12 QOL assessment form [25].

• All cause mortality

• Retention

### Duration

The trial will run for six months, with outcome assessment at baseline, 3 months and 6 months.

### Sample size

The sample size calculation is based on the test of the null hypothesis that the rates of adherence to HAART in the two groups (intervention and control) are equal. The primary measure of effect is the rate of adherence to ART treatment as measured by using the VAS over 6 months. The criterion for significance (alpha) has been set at 0.05. The test is 2-tailed, which means that an effect in either direction will be interpreted. The sample size was calculated using the WINPEPI (PEPI- for-windows) version 9.5 software [26]. With the proposed sample size of 82 in each of the two groups (i.e. assuming a 1:1 allocation ratio), the study will have power of 80% to yield a statistically significant result using a chi-squared test (assuming an intention-to-treat principle for the analysis) of the relative risk at alpha = 0.05. This computation assumes an adherence rate of 80% (for the intervention group) versus 60% (for the control group) at 6 months. These estimates are reflective of estimates from similar studies investigating SMS effect on drug adherences [27] and were modified to account for the type of intervention and patients for this study. We adjusted the sample size for a potential attrition rate of 20% (due to drop-outs) based on attrition rates to care in this centre. Therefore, the required sample size is 198 patients (99 per group). At the YCH ATC, on average, there are about 120 patients put on HAART per month. We estimate that about 90% will have mobile phones and would be eligible to participate in the study. Of these, it is expected that approximately 75% would be willing to participate in the study and will provide consent to participate in the trial. The expected period for recruitment will be one month to obtain 198 patients needed for the trial. It is feasible to recruit 198 patients in one month because we will also recruit from the large pool of old patients. Our study is designed to detect a 20% increase in adherence.

### Analysis plan

The analysis and reporting of the results with follow the CONSORT guidelines [28]. The statistician/data analyst will be blinded to the study group. The process of patient selection and flow throughout the study will be summarized using a flow-diagram. The analysis results of patient demographics and baseline outcome variables (both primary and secondary) will be summarized using descriptive summary measures: expressed as mean (standard deviation) or median (minimum-maximum) for continuous variables and number (percent) for categorical variables. We will adopt an intention-to-treat principle to analyze all outcomes, meaning that data from participants will be analyzed according to the group to which they were randomized even if they do not receive the allocated intervention. We will also use multiple-imputation [29] to handle missing data. We will use the T-test for comparing groups on continuous outcomes and the chi-squared test for binary outcomes. All statistical tests will be performed using two-sided tests at the 0.05 level of significance. The Bonferroni method will be used to adjust the level of significance for testing for secondary outcomes to keep the overall level at alpha = 0.05. For all group comparisons, the results will be expressed as effect (or risk ratio for binary outcomes), corresponding two-sided 95% confidence intervals and associated p-values. P-values will be reported to three decimal places with values less than 0.001 reported as <0.001. Adjusted analyses using the following baseline covariates (age, gender, education, duration on HAART, HIV staging, nutritional status (BMI) and the presence or not of an opportunistic infection (OI)) will be performed using regression techniques to investigate the residual impact of key baseline characteristics on the outcomes. Goodness-of-fit will be assessed by examining the residuals for model assumptions and chi-squared test of goodness-of-fit. All analyses will be performed using SPSS (Statistical Package for the Social Sciences) version 16.0 for Windows.

Adherence will be measured both as a continuous outcome (change in adherence) and as a binary outcome i.e. adherent (95% of pills taken) or non adherent (< 95% of pills taken). In literature, adherence data can be handled in a number of ways. The measures can be reported as the number of doses respected or can be combined into a composite score [21]. Even though combined measures are more correlated to clinical response, they are not very practical [22]. The data from the various adherence measures will not be merged. We will report the effects of the intervention on all the measures of adherence used, and compare them for discrepancies.

### Additional studies

• **Adherence rates: **We will use this opportunity to calculate the rates of adherence to HAART in the YCH ATC. The context of adherence to HAART in Cameroon has changed greatly over time and the rates reported in literature were subject to cost (which has been dropping over time), availability, accessibility, study design, technique used to measure adherence and study setting. These rates vary from 10% to 97% [30-32]. This study will provide a more accurate and current estimate of adherence in Cameroon.

• **Safety: **Data will be collected on issues that may arise from the use of text messages to improve adherence.

• **Health worker experiences: **Self-administered questionnaires will be used to assess health worker perceptions of the intervention in terms of long term use, additional workload and benefits to care. The applicability of such an intervention will depend largely on its acceptability by health workers.

### Ethical Considerations

The trial will be conducted in compliance with the local protocol and applicable regulatory requirements in Cameroon. The study has been approved by Cameroon National Ethics Committee. Any deviations from the protocol will be reported and explained. The study will be conducted in accordance with the Helsinki declaration [33] and other established clinical practice guidelines for research on human subjects. Research personnel will approach all potentially eligible patients who fulfill eligibility criteria for consent. All patients must sign a consent form to participate in the trial.

## Discussion

The potential of mobile phone technology to improve health outcomes is a domain worth exploring, especially in this era of increased uptake and dependence on mobile phones. Studies investigating the use of text messages to improve adherence have yielded varied results [12,16,34].

Improving adherence to HAART can play a key role in reducing morbidity and mortality due to HIV, the occurrence of drug resistant strains and the waste of medication in health systems that are already seriously challenged by the advent of HIV. Findings generated from this trial may be generalized to other chronic illnesses.

A major ethical consideration is the harm that may arise due to accidental disclosure of status. This eventuality will be properly explained to the participant, even though our message will neither disclose status nor medication. In a study evaluating the use of mobile phones to improve attendance to an HIV clinic in Uganda, privacy and confidentiality were not a problem [35]. Loss of privacy was not identified as a deterrent in the HIVIND trial [8,36]. We are aware that most mobile operators in Cameroon deliver mass text messages to their clients for business, advertising, information and sometimes health. Our intervention will be an addition to an already existing system. Another concern is how to manage text messaging on a larger scale, knowing fully well that it cannot be left in the hands of mobile operators, who will then be privy to the phone numbers of potentially identifiable individuals. In this trial, the operators will not be used because a framework for cooperation that will not jeopardize confidentiality and anonymity hasn't been established. A single phone with prepaid airtime will be used to deliver the text messages.

This intervention centers on the Health Belief Model of behavior change [37]. We will collect data on modifying factors (socio-demographic and disease related), perceived barriers to adherence and test the efficacy of the SMS reminder as a cue to action. Putting all these in context will provide a better picture of who doesn't adheres to HAART, why they don't adhere and if text messages can help. Applying the SMS not only as a reminder, but as a cue to action permits it use to resolve intentional and non-intentional non-adherence. In addition, we will open channels for communication between appointments. Galovotti et al succinctly describe the key components of an intervention aimed at bringing about a change in behavior. The use of role models, affective impact, and links to socio-cultural narratives, personalization and knowledge of impediments and facilitators are important features of successful behavioral interventions [38].

Finally, this trial may contribute to the growing body of evidence on the use of mobile phone technology to improve health outcomes in low resource settings.

## Abbreviations

ATC: Accredited Treatment Centre; BMI: Body Mass Index; CD: Cluster Designation; CIHR: Canadian Institutes for Health Research; CONSORT: Consolidated Standards for Reporting Trials; CTN: Canadian HIV Trials Network; HAART: Highly Active Anti-Retroviral Therapy; HIV: Human Immune Deficiency Virus; MEMS: Micro-Electronic Monitoring System; OI: Opportunistic Infections; PRD: Pharmacy Refill Data; QOL: Quality of Life; SMS: Short Message Service; SPSS: Statistical Package for Social Sciences; SR: Self Report; VAS: Visual Analogue Scale; WHO: World Health Organization; YCH: Yaoundé Central Hospital.

## Competing interests

The authors declare that they have no competing interests.

## Authors' contributions

LM conceived of the study. LT, POZ, RTL, EM, JV helped to draft the manuscript. DY, MJE, RCBM, RM, JSN, FN and HAO participated in its design and helped draft the manuscript. All authors read and approved the final manuscript.
